# Pneumonia

**Published:** 1854-08

**Authors:** 


ART. V.—*Pneumonia: its supposed connection, Pathological and Etiological, with Autumnal Fevers; including an Inquiry into the Existence and Morbid Agency of Malaria.* By R. LA ROCHE, M. D., Member of the American Philosophical Society; of the American Medical Association, &c., &c. Philadelphia: Blanchard & Lea; 1854.

---

THIS is a work of great research and great learning. Its author is a man of sound judgment, deep erudition, and cultivated taste; at once an observer and a thinker; with patience to collect facts, and a talent for philosophical classification and deduction. We have been waiting, up to this time, for leisure and space to notice it in a manner worthy of its high merits, and propose now to devote a considerable portion of our present number to an analysis of its contents.

Dr. LA ROCHE disclaims, at the outset, any pretensions to originality in the views set forth in this volume. He claims, not to offer anything new, but to have "collected, within a comparatively small compass, the main facts bearing on the question at issue." We agree with him, that much discredit has been brought upon the profession, and not a little injury done to the science, of medicine, by a straining after novelty, and by unfounded claims to originality. With teachers of a certain stamp it has been much the custom—more in former times, we think, than now—to set up on all occasions to be the expounders of new doctrines. They were "nothing if not *original*." Their lectures consisted of expositions of their peculiar views of medicine and medical philosophy. To present the science as others held and taught it, was unworthy of their genius; they aspired to be innovators—the founders of new systems. "On this subject," says Dr. LA ROCHE, "my mind has long been made up; and from all I have seen, I can entertain no doubt, that more good is to be effected by a patient accumulation and comparison of important facts, and by endeavoring to draw from the whole correct philosophical deductions, than by adopting a different course, too common among the professional writers of this country and elsewhere; who discarding the results of the experience of former and present times, and relying exclusively on their own, too often scanty, observations, make up for their other deficiencies by an indulgence in theoretical explanations; and sneer at the patient, slow, and cautious observer, and the erudite student."

To the latter unambitious class the author of the work before us is content to belong. What he has attempted in this volume is, to prove that the idea of the identity of pneumonia and autumnal fever "is founded on insufficient and incorrect data, and is, in fact, little more than a dream of the imagination;" and, at the same time, to demonstrate that there is good ground for the general belief in the existence of aerial poisons as the cause of autumnal fevers.

He begins by showing, that the belief in the connection of pneumonia with autumnal fevers has long been entertained, affording the modern advocates of the doctrine an opportunity to see "that they are treading on ground already and frequently traveled over and as frequently abandoned." LANCISCI, SYDENHAM, CLEGHORN, RUSH, MORTON, SAUVAGES, ALIBERT, BAILLY, and many other writers of note, would seem to have believed in this relation of these diseases, and they rested the opinion mainly on the fact that the two complaints "prevail simultaneously in the same localities, or succeed each other; and that while pulmonary inflammation often occurs in summer and autumn, when fevers are rife, so the latter appear also in winter and spring, when the former usually prevails extensively."

Having stated what is held by the modern advocates of this old doctrine, he proceeds thus to present his own views on the subject:

"I have no hesitation in stating, as the result of my personal observations aided by extensive inquiry and close reflection, that pneumonia is not *sometimes* but *always* an idiopathic disease, whether it occurs sporadically or epidemically; whether in the South, in the North, in the East, or in the West; whether in fever districts, or in fever seasons, or in places or at periods of the year free from periodical fevers; and that it is due to causes perfectly distinct from those to which such fevers owe their origin. I believe this; and believe, besides, that even were pneumonia produced at times by the legitimate causes of fevers, such cases would not be any more symptomatic than those that are due to the ordinary causes of the disease; for these do not act directly and primarily on the lungs any more than the others would do, supposing them capable of giving rise to the effect in question. And as in either circumstance the primary impression is received by some other part of the system, and thence reflected on the pulmonary organs, the two sorts of cases must be placed on the same footing, and be all primary or all secondary. Of course we must exclude from the cases thus referred to, those produced by traumatic or mechanical injuries, and agencies acting directly on the lungs, for these might, strictly speaking, be called symptomatic. It may, perhaps, also be proper to exclude some of those in which symptoms of pneumonia show themselves during the progress of other diseases, or after extensive surgical operations or wounds. I say in some, for while in these the inflammation of the lungs is secondary, and merely the effect of sympathetic irritations, which may and do arise in a variety of dissimilar complaints, and are somewhat influenced by the nature of the cause giving rise to them; in others, the pneumonia may properly be called idiopathic, inasmuch as it is independent for its causation of the agency giving rise to the prior complaint, being produced by the same causes which would have given rise to it had no such disease, operation, or wound preceded. But, whatever be the mode of origin of such cases, many of which properly belong to a category to which attention will be called by and by, it may safely be maintained that pneumonia, whenever it shows itself *ab initio*, is idiopathic, that it is always produced by its appropriate causes, and, what is more to our present purpose, that it is independent for its origin of those morbid agencies to which autumnal fevers are due; that it is not less independent of such fevers in a pathological point of view, and that it cannot, therefore, be lowered to the rank of a peculiar form of that class of diseases."

The facts upon which he relies in support of this opinion are the following: that pneumonia is common where fevers are seldom or never known; that it is not necessarily present where fevers are common; that the two diseases prevail at different seasons; that they appear under the influence of opposite winds; that pneumonia is of yearly occurrence, while fever is not always so, even in countries subject to malarious diseases; that their altitude of range is not the same, fevers being limited by elevation, while pneumonia prevails at all heights; that fevers are influenced by the nature of the soil, while pneumonia is a disease of all localities; finally, that frosts arrest fevers but not pneumonia. These are all matters of common observation and hardly need to be supported by the citation of authorities. In view of their unquestioned reality, Dr. LA ROCHE seems justified in using the following language:

"So far as they go, they seem conclusive; and with suitable deference to the authority of writers who entertain sentiments different from those which it is my object to uphold, I cannot think I hazard much when expressing the opinion that the impartial reader, after perusing what precedes, will acknowledge that it will puzzle them, talented and ingenious as some of them undoubtedly are, to reconcile those facts with the hypothesis set forth regarding the identity of pneumonia with malarial fevers. Let them, if possible, account for the aforesaid circumstances—the absence of malarial fever where pneumonia is rife; and the extensive prevalence of the former in localities where the latter is either not of remarkable frequency, or scarcely seen at all—the cessation of the one and continuance of the other—and say how all this could be brought about, if the inflammation of the lungs, in the disease in question, were due to the agency of the cause producing those fevers; in other words, if pneumonia were really and substantially nothing more than a peculiar form of remittent and intermittent fever. Were the etiological connection and pathological dependence such as maintained, we should expect to find that the cause, if diffused in such localities to an extent, and possessed of a degree of energy, sufficient to produce a large number of cases of pneumonia, or, as others would say, of the pneumonic form of autumnal fever, would also give rise to a greater or less number of cases of the other and more legitimate and characteristic forms of those fevers. This would be the more natural to anticipate, because the climatorial influences noted in those favored spots where fevers have never prevailed, or have long ceased to do so, are not different from those under the empire of which such fevers everywhere appear; or have remained the same amid all the changes that have occurred in regard to their prevalence;—the only difference consisting in the absence or removal of certain terrestrial or local conditions, which, whatever be the nature of their association with the ordinary forms of the supposed protean disease, have apparently nothing in the world to do with the existence of what is now maintained to be simply another form of the same. The result being different—pneumonia occurring where autumnal fever has never originated, or where, if it has done so, it now seldom if ever prevails—we are warranted in concluding that the causes of the two diseases are different; that the one may exist without the other; that when the two diseases show themselves at the same time, and in the same locality, two sets of causes necessarily exercise their baneful influences and produce, not one disease assuming different forms, and presenting different aspects, but two distinct complaints; and that, consequently, pneumonia cannot justly be held up as forming part and parcel of autumnal fevers, which, as regards etiology, are governed by very different laws, and influenced by very different agencies."

To the majority of his readers the arguments of Dr. La Roche adduced in the first hundred pages of his work will appear perfectly conclusive, and for them the discussion might have ended with his first chapter. In fact, there are many who regard any argument against the hypothesis he is opposing as unnecessary, and he has been charged by some of his reviewers with assailing what no one supported. But this is a mistake. The doctrine has its advocates, to whom the elaborate argument of this work is by no means satisfactory; and there are also those who question the existence of any specific cause of fevers, but hold to their having their origin in atmospherical vicissitudes. Dr. La Roche, therefore, before proceeding with the discussion immediately in hand, takes up this question and devotes three chapters to its consideration.

It is a full, earnest, and most able presentation of the subject. Beginning with the history of scientific medicine, Dr. La Roche shows that the belief in an extraneous unwholesome principle in the atmosphere as the cause of fevers, has had an existence in all ages. Hippocrates held that the cause of epidemic fevers resides in the air, and he, with great acumen, accounted for the fact that all animals are not alike affected by this äerial poison, upon the principle "that what is proper or improper for one species is not necessarily equally so for other species." Antipater, the Stoic, speaks "of exhalations from the damp earth, that dart down again like deadly serpents, poisoning and infecting every thing by means of their corrupting agency." The Romans had equal faith in the reality of malaria, and their poets refer it to the joint action of the sun and stagnant waters. Columella cautions against the erection of houses or military posts in the neighborhood of marshes, because when acted upon by heat, he says, they eject a baleful poison, giving rise to "obscure diseases;" or in other words, to autumnal fevers. Palladius, in like manner, and also Vitruvius, speak of the danger of residing near to marshes; and Diodorus Siculus gives an instructive example of the perils of such sources of inefction. At the siege of Syracuse, he states that "the Carthagenians being encamped in the immediate vicinity of an infectious marsh, and exposed to the thick and heavy vapors issuing therefrom, and being moreover accumulated on a low and humid locality, a pestilential fever broke out among them, and gave rise to a large mortality." To the same cause this writer ascribes the plague of Athens. But it is not our intention to follow Dr. La Roche in his history of medical opinion on this subject. We will simply remark in leaving it, that no writer in our country is better qualified to do the subject justice, if indeed there is one whose mind is so fully stored with all the learning that bears upon it.

He takes up the various arguments urged against the agency of malaria, and his answer to each seems to us fair and satisfactory. The mass of facts which he has accumulated on this point is immense, and the conclusion to which they will bring any unbiased mind, we think, can hardly be doubtful. All that we can do is to select a few of his examples, one of which is as follows:—

"If we approach to, or remain some length of time—occasionally a few hours or moments—in those localities, or in their immediate vicinity, we are stricken down with fever; if we avoid them we escape. The South Carolinan gives up his plantation residence in summer, and removes to Charleston or to the mountains, where he is safe from the country fever. Let him visit his estate before the advent of frost, and especially let him sleep there, and he runs great risk of an attack. In yellow fever seasons, strangers must leave or abstain from entering the city ; if they venture into it, they will in all probability have the disease. With us in Philadelphia—as with the residents of other cities of the middle and neighboring States, and of some parts of Europe—where infected districts are of limited extent, the disease is restricted to individuals who venture within the bounds of these. At a very few paces from the sickly spot to which they penetrated, and where they doubtless imbibed the seeds of the fever, people move about, business is transacted with impunity, and every thing often looks precisely as if the city were not the seat of an epidemic. By avoiding our river banks, or our meadow or marshy land, by remaining within the limits of the city, or selecting high and dry situations in the country, Philadelphians, like Charlestonians, keep free from chills and fever, or remittents ; by adopting a different course, they expose themselves to an attack. On the coast of the West Indies, and of Africa, as well, indeed, as in this country and Europe, vessels remain healthy so long as they keep at a distance from land.* But woe to them if, during the sickly season, they approach the shore, or enter the river streams. The moment they do that they become liable to the disease. Lind, in his work on preserving the health of seamen, states that when Commodore Long's squadron, in the months of July and August, 1744, lay off the mouth of the Tiber, it was observed that one or two of the ships which lay nearest the shore began to be affected by the pernicious vapor from the land ; whilst some others, lying farther out at sea, at but a very small distance from the former, had not a man sick at the same time."

It is contended by some etiologists that vicissitudes of temperature are sufficient to explain the rise of fevers. To this doctrine Dr. L. opposes the following objections :—

"But after carefully examining what the advocates of the opinion have adduced in its support, it appears to me that were atmospheric vicissitudes the efficient agent in the production of periodic and other forms of malarial fever, we might expect to find these diseases prevailing principally in seasons in which the number of dew or cold nights following on hot days is greatest.

---

* Lind, on Hot Climates, 138–178–9 ; *Ibid.* on Seamen, 78 ; Trotter, i. 456 ; Rouppe, 75—English translation, 69 ; Rush, iii, 83 ; Bancroft, 171 ; *Ib.* Sequel, 166; Clark on Long Voyages, i. 124 ; Moseley, 57 ; P. McLean, 26 ; Ferguson's Recollections, 151 ; Gillespie, 20 ; Fontana, 12 ; Bally, 455 ; Pringle, 57, 98 ; Chervin's Letter to Dr. Manfalcon, 12 ; Burnett, 264–274 ; Caldwell's Prize Diss. 139 ; Caldwell's Essay on Mal. in Boston Jour. 510 ; Williams's Morbid Poisons, ii. 446 ; Smith, Edinburgh Jour. xxxv. 49 ; Amie, Edinburgh Jour. xxxv. 264 ; Johnson, Charleston Jour. iv. 160.

We should be justified also in expecting malarial fevers to occur not once or occasionally, but frequently, if not universally, whenever the supposed cause manifests itself; or, rather, it ought to be found that every time such fevers prevail—sporadically or epidemically—the difference of temperature between day and night is greater than in healthy seasons. We should besides expect to find them appearing, not sporadically only, but in an epidemic form, as well in clean, well-paved, and well-aired cities, where atmospherical vicissitudes are as apt to be felt as elsewhere, as on the borders of creeks, rivers, and lakes, and in meadow lands, in level plains or marshy localities; and whenever a man whose body has been overheated is suddenly exposed to a cold atmosphere, or plunged into a cold bath, he ought to be regarded as no less liable to suffer from an intermittent, a remittent, or yellow fever, of the most legitimate kind, than from a pleurisy, a catarrh, or any other kindred disease. But so far from this being the case, experience shows that fevers occur and prevail extensively in situations where, and at periods when, such vicissitudes are not felt at all, or are so to too inconsiderable an extent to be productive of the baneful effects ascribed to them; and, on the other hand, that those diseases are either seldom felt or completely unknown in localities where, or seasons when, sudden changes of temperature, or the contrast between night and day, are as common and noted as, if not more so, than in places, and at times remarkable for insalubrity. Surely a morbid agent which, if it really exercises any influence in the production of autumnal fevers, does so only in localities of a special kind, where, let it be remembered, those fevers often appear and even abound without its aid; an agent which habitually fails to produce those same fevers in localities of a different kind; which produces the effect ascribed to it only in a certain season of the year, however manifestly it may show itself at other periods, and whose known ordinary products everywhere and at all times, are diseases very different in every respect from those in question; such an agent, I say, cannot lay a just claim to be held in the light of the efficient or necessary cause of the latter.

"The climate of Newfoundland is marked by sudden and frequent vicissitudes of atmosphere; notwithstanding which, however, fevers are scarcely known there, even among those most exposed.* The whole coast of this continent, from one extremity to another, is proverbial for the frequency, suddenness, and extent of such changes; and yet, while some localities are annually the seat of the pyrexial complaints under consideration, others, equally subject to the former, remain untouched by the disease."

It is a matter of general observation, that fevers are not always

* Tullock, Sickness, etc., of British Army in British America, 35.

generated in places which seem to combine all the circumstances favorable to their production. In the midst of marshes we sometimes find the inhabitants, for a time, enjoying an immunity from fevers. The exemption is to be explained in various ways:—

"Sometimes it is due to the high elevation above the level of sea of the places so exempted. At another time, the effect is attributable to the absence of a sufficiently high and long continued atmospheric heat. In other instances, the circumstance is due to a very perfect and constant ventilation, and a very rarefied and pure character of atmosphere. In some instances, again, it may be explained by the peculiar geological character of the soil; the quantity and the quality of the surface-water; or the proportion of sulphates the latter contains in solution. Sometimes, also, it is due to the rapidity of the river currents, the excessive and rapid dryness of the atmosphere during the hot season, the existence and extensive prevalence of refreshing and purifying winds, and often to the degree of desiccation the surface has attained by natural or artificial means, the degree of cultivation to which it has been carried, and other agencies of like import, as well as by the extent to which it is sheltered, by rich foliage and other means, from the action of the sun. So far as ships are concerned, the freedom from fever will often be found ascribable to the latitude in which they may be navigating, to the early period of the year in which they may be at sea, or otherwise employed; or to the absence of an epidemic constitution of atmosphere.

"In the examination of the subject, none of these contingencies should be overlooked. Experience has shown that there is an altitudinal range, varying in different parts and according to the peculiar form of the disease, beyond which, owing to the greater rarefaction of the air, peculiarity of temperature, or other circumstances, the elimination of the febrile poison does not take place, or the latter is rendered inert; that a certain range and continued elevation of the thermometer is required for its development; and that free ventilation, and strong unimpeded currents of wind are inimical to its morbid agency. Experience has shown in addition, that while, as we have seen, an argillaceous soil is prone to the development of fever, a region of primary formation, with a sandy, calcareous, arid, and sterile soil, allowing no stagnant water, and containing only a small proportion of organic remains, is usually exempt from the disease; that, in not a few instances, the passage from a fever to a healthy locality takes place within circumscribed boundaries, and is indicated by a difference in the geological formation of the soil; and that, if exceptions to this occur, the explanation is easily found in the fact that the cause of the disease has been wafted from some pestiferous region, to a locality which otherwise, would have remained unaffected; or in the circumstance that the calcareous structure of the sickly spot, though naturally of a kind not subject to fever, is covered over, extensively or in spots, with a more or less thick coating of rich absorbent soil, possesses an argillaceous substratum, or presents depressions, in which water stagnates, and the process of decomposition takes place. Again, experience has shown that water containing a small amount of sulphates, is less injurious to health, and less prone to favor the formation of malaria, than that which is richer in those materials; while on examination, it is found that the vegetable matter contained in peat moss is subcarbonized, and necessarily unsusceptible of decomposition; that such moss as already stated, is known to possess peculiar antiseptic qualities, which, by imparting to it the power of preserving not only trees and other vegetable, but animal substances from putrefaction, renders it unfit to evolve the efficient cause of periodic fevers.

"See the remarkable contrast noticed in the relative prevalence of agues in New England, New Brunswick and Nova Scotia, on the one hand, and on the other, in the region of the great lakes on either the American or British soil. This contrast, resulting from the absence of such fevers in the former region, and its great prevalence in the latter finds no explanation, as Dr. Forry well remarks, in any difference of climate as regards temperature and moisture; but the solution must be sought in the modification of climate arising from geological formation and the nature of the soil. "Now, as the region of New England, as far as the St. Lawrence, with little exception, has a primative formation, with a sandy, sterile soil, whilst that of the lakes consists of a secondary formation, having not unfrequently, an alluvial superstratum, composed of a rich vegetable mould from three to six feet deep, it is not difficult to deduce the correct inference. In the former, the geological structure is destitute of organic remains, and the little contained in the sandy soil does not find enough moisture to induce the necessary chemical action; while in the latter, not only is the geological formation of secondary origin, but the deep, rich soil is sufficiently humid, when a high temperature acts upon the organic remains with which it abounds, for the development of the morbid poison called malaria."

"The fact that some marshy surfaces, which are supposed to give rise to fevers by means of the exhalations issuing from them, prove completely, or to a great extent, innocuous in certain seasons, and even during a succession of years, cannot be urged as an argument in favor of the denial of the malarial cause of fever, finding, as it does, a ready explanation in the absence, during those seasons of exemption, of the thermometrical and hygrometrical conditions, which experience has taught us to regard as essential to the evolution of the poison, as well as, often, in the absence of that peculiar state of atmosphere to which the name of epidemic, constitution, or meteoration has been applied; and the agency of which, in diffusing and enhancing the virulence of the cause of certain diseases—explain it as we may, and extraordinary as it may appear—it would be just as reasonable to doubt, as to doubt the existence of fevers themselves."

Malarial fevers unquestionably depend upon a number of concurring circumstances, and if any one be wanting the disease will not arise. Marshes in high northern regions are innocuous, and a certain elevation is equivalent to a number of degrees of latitude. As we ascend elevated mountains we virtually, as to fevers, pass into northern regions. And so the degree of heat, shelter from the action of the sun, ventilation, and humidity of the soil are all agents of a controlling or modifying character, too much or too little of the first and last producing the same result. There must be a certain amount of heat and moisture to induce decomposition of organic matter, which is arrested alike by the excess or the absence of either.

"Now, when we find the cause of fever requiring for its development the action of the very agencies which are necessary to insure the development of the gaseous products of decomposition—when we find that without these agencies, applied in certain proportions, neither those gaseous products nor the efficient cause of fever will manifest themselves; and that in all instances in which the latter is produced, as shown by the occurrence of fever, materials capable, when acted upon by the agencies in question, of giving rise to the evolvement of the gaseous products of decomposition—organic matter in various conditions and states of modification exist; and that the total absence of these materials—whatsoever be the degree of heat, and of terrestial and atmospheric moisture—carries along with it an absence of fever, we can have no reason to doubt the propriety of admitting that the cause of the disease bears a close analogy to the aforesaid gaseous products; and that if in regard to the former, heat, humidity, and other agencies, acting in given proportion and in concert, on animal or vegetable matters, give rise to the evolvement of certain gaseous substances, the febrile poison, which in like manner requires for its development the action of the same agencies, as also the existence of kindred organic matter, must necessarily consist also of some modification of a similar kind of gaseous substance."

Dr. La Roche continues through the two succeeding chapters to multiply proofs of the existence and morbid agency of malaria. His collection of facts in support of his position is immense. No authority seems to have escaped him, and if he has not succeeded in establishing the point in question, no future effort short of the demonstration of the poison promises to avail anything. The details into which he enters, although extended, are by no means tedious, but, on the contrary, will be read by those whose minds are in doubt on the subject with sustained interest. The learning displayed by the author in this discussion, the patience of research, and the calm, philosophical spirit in which it is conducted, are worthy of the highest praise. As we have accompanied him through the argument, we have been continually reminded by his abounding treasury of facts of the labors of another honored American physician—of a volume which he, in accordance with general opinion, pronounces "imperishable." In many points, this work of Dr. La Roche's bears a decided resemblance to the treatise of Dr. Drake, and must take its place in the same rank with that monument of industry and learning. We shall not quote any further from this part of his volume, but return to the comparison of pneumonia and autumnal fevers, which he resumes in his fifth chapter.

In the first place, he shows that the causes of these fevers and pneumonia are different. Thus, malarial fevers are restricted within certain localities, often very limited. Further, the cause of these fevers is wafted by the wind, so that places in the neighborhood of marshes and ponds, but in opposite directions from these sources of malaria, are differently affected, accordingly as the winds bear the poison to them or carry it away, neither of which can be asserted of pneumonia. And again it is well known that overflows of the sea, lakes, and rivers; the establishment of mill-dams; the clearing of new land; the digging of canals; the partial draining of marshes, all are fruitful sources of fever; but are harmless so far as thoracic inflammations are concerned. Numerous other points of difference might be pointed out, but these are deemed sufficient.

Pneumonia is a disease of all climates and all seasons, while autumnal fevers, as the name implies, are especially prevalent at certain seasons, and are localized in certain places.

"Let us examine the subject in another point of view, and acknowledge, for a moment, that remittent, intermittent, and yellow fevers, and other zymotic diseases more or less liable to them, are not the offspring of peculiar morbid poisons exhaled from the localities where they prevail; and are, so far as that goes, on a par with pneumonic inflammations; I am not sure that the admission would afford much help to those whose opinions are under examination; for it is impossible to shut our eyes to the fact, that the localization of those diseases takes place only where certain peculiar combinations of materials appertaining to the soil, or which have found their way there accidentally or otherwise, exist. Bearing this in mind, we arrive at once at the conclusion, that the real cause, whatsoever it may be, meets there certain agencies which so modify the system as to render it liable to their morbid impress. In a word, what many regard as the active and efficient cause of those diseases, may, after all, be only a predisposing agent. If this be correct, autumnal and periodic fevers are, in that respect, on an equal footing with other zymotic diseases arising from specific ferments or poisons. Every one knows that while Asiatic cholera and the febrile exanthemata are never produced by the malarial exhalations evolved frem foul localities or marshy surfaces; while typhus and typhoid fevers are, as it is said, seldom the offspring of the former, and certainly never of the latter; while none of these diseases are occasioned by the ingestion of putrescent food, by the use of foul water, by imperfect ventilation, by starvation, by excessive muscular exertions, by the intemperate use of alcoholic liquors, and the like; and while, with the exception, perhaps, of typhus, they do not arise from the effluvia proceeding from the human body—particularly the lungs and skin—and consisting of the effete and highly putrescent matter mingled with the air or prespiration—it is a notorious fact, that they are principally rife in situations where such influences operate, and strike with greater violence, malignancy, and fatality among individuals exposed to their baneful effects. This is true, whether the disease be the product of a zymotic poison floating in the atmosphere, and independent for its development of any organic process, as Asiatic cholera; or whether it arises from a poison formed in the system and transmissible from one individual to another through means of contact, or the medium of the atmosphere; or whether, again, it is due to a particular poison proceeding from external sources of animal or vegetable decomposition, or from the result of a morbific condition of the system, as is the case, perhaps, with puerperal complaints, erysipelas, and *surgical fever.*"

It is hardly logical to infer, that diseases differing so widely as pneumonia and malarial fevers both in symptoms and anatomical lesions, are the products of a common cause. We conclude that small-pox and measles, that yellow fever and intermittent fever, that cholera and influenza, are engendered by different causes, because in symptoms they are so different from each other. Pneumonia and malarial fevers are as unlike each other as are any of these diseases, and moreover nothing is better understood than that the fever of pneumonia is entirely dependent upon the local inflammation, and yields when the inflammation is subdued. The phenomena of pneumonia are those which indicate acute inflammation; in fever the inflammation, if it supervene at all, is secondary. The pathological conditions and results are different.

"It need scarcely be remarked, that in all inflammations the blood is in what has been denominated a state of hyperinosis. It contains more fibrin than in the normal state—5 or 6 parts in 1,000, instead of 2 or 3—while the corpuscles decrease in proportion to the excess of the fibrin, from about 141.1 in 1,000 to about 128.0. The fatty matter also is increased; and, as a consequence of all these changes, the whole solid residue is diminished. The blood coagulates more slowly than in the normal state; the clot is not usually small, but very firm and consistent, and does not break up for a considerable time. It is almost invariably covered with a buffy coat, which is firm, tough, and intimately connected with the clot; its edge is often turned upwards and its surface uneven. If the clot be small, the buffy coat and the surface of the clot are more or less cupped, and the serum is of a pure lemon color, not tinged red. When whipped, the fibrin separates in thicker and more solid masses than in ordinary blood. After the removal of the fibrin the corpuscles quickly sink, and frequently occupy only one-fourth of the whole fluid, while in healthy blood they sink very imperfectly, or not at all. The blood has always an alkaline reaction, and is of a higher temperature than in the ordinary state. All these changes are proportionate to the degree of the inflammation. Such is the condition of the blood in the phlegmasiæ generally. In pneumonia, the state of hyperinosis is even more decided than in other diseases of that class, as the blood retains its heat longer; the clot is below the ordinary size, and very consistent, and does not break down for a considerable time. It admits of being sliced, and the sections retain their consistency for some time. Its surface is covered with the buffy coat, and is more or less cupped. The serum is of a pure yellow color. The quantity of solid constituents is usually less than in healthy blood. The proportion of fibrin increases and varies, according to Andral and Gavarret, from 4 to 10.5 in 1,000 parts, with a mean (in 58 experiments of 7.5; from 5.919 to 12.726, according to Rindskopf, and 3.4 to 9.15, with a mean of 6, according to Simon; while the blood corpuscles, according to the first experimenters, amount to from 83.2 to 137, with a mean of 114.1; and, according to Simon, to as little as from 36 to 78. If other symptoms present themselves, or if the blood assumes a different appearance, these results are due to complications; they do not constitute a necessary link in the chain of phenomena imparting an individuality to the disease, and are not, therefore, pathognomic."

The blood in fever, on the contrary, is of a soft, diffluent, and dark color, without buffy coat, of an alkaline reaction, sometimes poor and watery, in some instances forming no coagulum, all going to show a deficiency of fibrin.

"Can any one, with such facts before him, seeing an increase of fibrin on the one hand, and a diminution of the same element on the other, unite in sentiment with those who regard the two diseases as pathologically identical, and maintain with them that pneumonia is really and substantially nothing more than a peculiar form of remittent and intermittent fever? In other words, can he be persuaded that a peculiar disease, characterized by a particular condition of the blood, and depending for its existence on the local inflammation of a special organ, is only a different form of another disease, marked by a diametrically opposite condition of that fluid, and not necessarily connected with the inflammation of that, or any other organ?"

The other anatomical characters of the two affections differ not less widely than those of the blood, and added to these circumstances, so convincing in their import, we have the fact that the diseases are different in the duration of their processes of incubation, and also that, while one attack of pneumonia rather predisposes to other attacks, a comparative immunity from some forms of autumnal fever is enjoyed by those who have once labored under them.

In the next chapter, Dr. LA ROCHE goes on to show that the power of acclimatization does not extend to pneumonia, as it is known to do to yellow fever, and that fevers and pneumonia are not alike in the force with which they assail the several races. Negroes, it is admitted, are far less susceptible to the poison of fevers than are the white race, but are quite as liable to attacks of pneumonia. And so in regard to age. Children are often the subjects of pneumonia, but fevers are emphatically diseases of adult life. Passion and the emotions influence pneumonia but little, while all observant practitioners have remarked the baneful effects of mental depression in the production of fevers.

But we must leave these points and proceed to give the author's views of the relations of pneumonia to autumnal fevers, which are summed up in the statement that, although these diseases are "independent of each other, as regards nature and cause, they combine together, and form, like other complaints, hybrid diseases."

The concluding chapter of this learned work is taken up with proofs confirmatory of this proposition, and we hesitate not to express our thorough conviction that this view of the matter is the correct one. In temperate climates, and particularly in malarious districts, pneumonia is unquestionably often a hybrid disease, periodical in its type, and demanding the remedies applicable to intermittent fever. Dr. LA ROCHE remarks:

"It is a fact well ascertained, and perfectly familiar to those who have investigated the subject of the progress and succession of epidemic or endemic diseases, that the type of the fever which prevails immediately before the outbreak of pneumonia—in other words, before the period of the year at which the usual causes of the latter are mostly encountered and operate with more force and effect on constitutions predisposed to their action arrives—impresses its own character on pulmonary inflammations. Hence at that season, low surfaces, the vicinity of mill-ponds, of the banks of streams, and of other localities, which before were the abodes of pure remittent and intermittent fevers, become the seat of pneumonias, which often assume a marked remittent, and not unfrequently an intermittent character—the result of anterior influences; while the same disease in other situations where malaria is not evolved, or at a more advanced period of the year, when it has been completely destroyed, presents nothing of the kind for a longer period. The same combination of phenomena is necessarily observed for a longer period whenever pneumonia shows itself in localities where malaria continues to be evolved all the year round, or where, from the absence of frost, it is only moderated, and not completely destroyed. In many such cases the inflammation of the lungs presents itself in combination with symptoms appertaining to ordinary bilious remittent fever, or hepatic or bilious derangement, giving rise to what is denominated bilious pleurisy, a form of disease accurately described by many American and European writers. In other instances, the bilious symptoms are not so prominent, and the affection of the lungs is associated with those of simple remittent or intermittent fevers. Indeed, instances of remittent and even intermittent pneumonia, pleurisy and pulmonary catarrh, in which the inflammation is complicated with symptoms indicating the existence in greater or less purity of the element of periodicity, are to be found described in the writings of the most reliable authors. Morton, who early called attention to them, and indicated the treatment they required, had seen a hundred such cases; and since his day they have continued to be adduced as objects of familiar professional observation, not only in Europe, but in this country also."

These facts have long been familiar to the profession, and the remark is as applicable to pleurisy, influenza and bronchitis, as to pneumonia. The pleurisies of Minorca, in 1745-6, were described by CLEGHORN as remittent or intermittent in their character, and Sir GEORGE BAKER describes the influenza of 1762 as a periodical disease.

"In an excellent essay on the 'relation existing between epidemic and other diseases prevailing at the same time and place, and denominated intercurrent,' Raymond, among others, in reference to facts he had observed during a long series of years, dwelled at some length on the complication of pneumonia with the epidemic or stationary constitution of the atmosphere existing at the time the disease happens to show itself, and remarked:—'Those kinds of thoracic inflammations have constantly assumed the types of the epidemic constitution during the existence of which they appeared. They have engrafted themselves on the epidemic or constitution of the year, and presented the same symptoms and the same functional lesions, in addition to the affection of the respiratory organs which characterizes them in a special manner. Apart from the expectoration, which appertains to them, their march, their critical movements, and their mode of termination, were the same. Inflammation of the lungs is, therefore, composed of, or complicated with, the stationary modes or symptoms of the constitution or epidemic of the year, and of the transient and intercurrent constitutions of the period and of the seasons from which they arise.' If, he continues, intercurrent diseases are founded on constitutional fevers, in their turn intermittent fevers are often complicated with the elements by which the former are characterized."

Dr. LA ROCHE further remarks in continuation of this subject:

"The true nature of these cases did not escape the sagacity of our great medical philosopher, Dr. Drake. 'The lungs, it is well known,' as he remarks, 'are liable to inflammation in this fever; and, instead of occurring late in the disease, like cerebritis, it generally arises at an early period. Such inflammation may prove fatal; and then a *post-mortem* inspection will show the lesions resulting from bronchitis or pleurisy, but more frequently still those of pneumonia, such as sanguineous engorgements and hepatization. But they cannot be regarded as constant, essential, or characteristic of autumnal fever; for, *first*, a vast majority of cases even those which prove fatal, do not present a single symptom of pulmonary inflammation; and, *second*, this inflammation, in most instances, is the undoubted effect of sudden changes of weather in the latter part of autumn, and must, therefore, be taken as the offspring of an incidental cause, acting subsequently to that which produced the fever.' In another place, Dr. Drake states that the most frequent of the complications occasioned by the influence of malarial fevers is that presented by the pneumonias of the south, and also of the lakes in the north.

"Again, in speaking of the complications of intermittent with other diseases, the same eminent writer farther says: 'but the more frequent and formidable of these complications is that presented by the pneumonias of the south, as also on the shores of lakes in the north, where numerous cases occur, which the profession too often find unmanageable by any method of treatment they have been able to devise.' In another place, he calls attention to the fact that the subdiaphragmatic viscera, except the pancreas, are subject to inflammation in remittent fever, and says: 'Sometimes, however, from idiosyncrasy, or the co-operating action of other causes, inflammation in other parts occurs,' 'and, when the fever makes its attack late in autumn, the combined action of vicissitudes of temperature, and that of the specific cause, developed at an earlier period, may determine inflammation upon the lungs or pleura.' 'The pneumonia bilioso,' says Dr. Potter, 'is a compound affection, originating from a double remote cause.' 'It is the immediate offspring of a low temperature, engendered upon a miasmatic predisposition.'"

The practice of administering anti-periodics in such hybrid cases of pneumonia is not a novel one in medicine, but we agree with Dr. La Roche that it is one which may easily be abused or misapplied. He says:—

"Now, as to the former of these points—the great benefit or superiority claimed for the quinia practice, when resorted to during the abatement of febrile excitement in pneumonia—I cannot greatly err, when expressing the opinion that many, very many practitioners, in all parts of the world, who have seen much of the disease, and acquired the needful skill in its management, will feel no disposition to join in singing the praise of that method, or to adopt it to the exclusion of the one they have heretofore employed. They will say, and I suspect that statistical returns will bear them out in the assertion, that while autumnal fevers, especially when they assume the truly periodic type, call, in some of their stages, for the use of anti periodics, and more particularly of quinia, it is very far from being proved that the same will hold good with regard to pneumonia and kindred thoracic inflammations, which are, to say the least, cured just as well without as with the aid of the salts of bark; that in their usual form they yield to antiphlogistics, general and topical, and to revulsives, or even, in some instances, to the powers of nature; that, in the majority of cases, the quinia practice would be not only useless but hazardous, and should on this account be avoided; that, as a general rule, the remedy, if used at all, can only be so after other means of a different or opposite kind have been resorted to; and that, when these have been properly timed and judiciously employed, the disease seldom requires the aid of tonics, still less of those possessing an anti-periodic power. They will say, besides, that when tonics are called for, it is not in virtue of their anti-periodic effects; that as much advantage, if not more, is derived from other articles of the Materia Medica; and that, when the disease assumes the typhoid form, it may be more successfully treated by local depletions and revulsives, or by the latter without the former, together with stimulating diaphoretics and expectorants, and sometimes with stimulants and tonics. Finally, they will say that, when the latter are required or admissible, quinia will often answer a good purpose; but that it is doubtful whether it will be more serviceable than other articles of the Materia Medica. In all this, the majority of professional men throughout this country and in Europe will, I have little doubt, acquiesce. In these parts, where, I should presume, the treatment of pneumonic inflammation is as well understood, and as successful, as it is in any other section of the world, I feel confident that few physicians of note would be disposed, from experience, to resort to quinia or other kindred remedies at any but the last period of the disease, or when signs of prostration or relaxation of the powers of life manifest themselves."

Dr. LA ROCHE is skeptical as to the high remedial virtues claimed by some for quinine in pneumonia. and regards the large doses sometimes prescribed as being attended with danger, in which opinion he is borne out by the experience of BRIQUET, BENNET, MELIER, DESIDERIO, GEROMINI, BALDWIN, and others. He quotes these writers to show that this article, when given in an over-dose, produces drowsiness, a difficulty of keeping the erect posture, a tendency to immobility, dimness of vision, cerebral excitement, and, when thrown into the blood-vessels, general convulsions. But in whatever doses given, many of those who formerly insisted upon its efficacy in pneumonia are now disposed to discard its use in this disease, except in those cases which are strongly marked by intermissions, and in which the malarial complication is clearly exhibited.

"In these, and in every other case in which this periodic element is manifest, quinia should, undoubtedly, be resorted to, more especially when the disease displays a pernicious or malignant tendency—a circumstance which renders the recurrence of a paroxysm of the utmost danger. These cases, in which the inflammation is in all probability modified by the malarial taint, in such a way as to tolerate the use of remedies, which, under other circumstances, could not be borne with impunity, resist every other mode of treatment. They cannot be cured by antiphlogistics alone; for although by these the inflammatory affection of the lungs may, if uncombined, be removed, the malarial fever or taint is not to be so destroyed; and the recurrence of every paroxysm or exacerbation, has the effect of aggravating the local disease; which cannot therefore, be eradicated, unless a stop be put, by anti-periodic remedies, to the complicating complaint. With the cessation of the latter, the pneumonic inflammation, if it has not reached beyond the first stage, generally disappears also. In other cases, it abates considerably, as indicated by an improvement in the general symptoms and physical signs."

Sarcone, as long ago as the year 1764, bore testimony to the value of this practice in such complications. "There is no one among us," he says, "who is not in a condition to present numerous observations respecting the happy results obtained by cinchona in affections of the lungs, combined with periodic fever. I myself, though young in the profession, am able to adduce numerous examples of the useful employment of that remedy in the diseases in question."

But the application of this practice in pneumonia is exceedingly limited, even in southern countries, there being only now and then a case of a well-marked periodic character to which it is adapted. It is only in such instances, where malaria is exerting a joint influence with the inflammatory disease on the system, that quinine is efficacious, and therefore the argument founded on the general superiority of this mode of treatment fails in this, that its truth is far from having been established. We are sure that few practitioners after a fair trial for a number of years would be disposed to claim for it a general application to pneumonia.

We make one quotation more from this excellent work, and with it must bring this article to a close.

"As to the second—that the success of quinia in pneumonia must be viewed as a sure proof that this disease is identified with, and constitutes only a particular form of periodic or autumnal fever, in the treatment of which that remedy is, if not a specific, at least a most efficacious remedy; it need only be remarked that, even were we disposed to recognize the validity of the claims set up in favor of quinia in all instances of pneumonia, it would be illogical to deduce from that superiority a proof of the identity in question. As everyone knows, quinia is daily used advantageously in diseases which owe their origin to causes very different from the legitimate poison of autumnal fevers, and which, nevertheless, it is not probable any one would be disposed to regard as constituting really and substantially anything more than particular forms of those fevers. In articular rheumatism, arising without malarial taint, and having nothing in the world in common with autumnal fevers, it has been and is employed by Briquet and others, with, as it is said, great utility. No one but Dr. McCulloch will be inclined to maintain the intimate and *constant* dependence of neuralgia upon intermittent fever, and to regard it as being produced *exclusively* by malaria. That it is often, as remarked by Dr. Drake, the *consequence* of autumnal fevers, especially when it assumes the periodic type, no one will deny. But it is also found to be the consequence of other complaints in no way allied to such fevers;—cases occurring and assuming the intermittent character under circumstances which forbid the idea of any malarial agency. Nevertheless, quinia is often beneficially employed in the various forms of that painful disease.

"Whatever be the cause of periodicity in a disease, or rather whatever be the nature and cause of a disease, which presents a well-marked remittent or intermittent character, quinia will be found a useful remedy in its treatment. It is useful also in certain nervous disorders in which the periodic element does not manifest itself, and which have no more to do with periodic fever than with smallpox or syphilis; and if we conclude that pneumonia, generally, is really and substantially nothing more than a peculiar form of remittent and intermittent fever, and should take its appropriate place (in company with pleurisy, &c.,) under the plain designation—*periodic fever*, on the ground that certain cases of it are, under particular circumstances, and at a particular period of their course, greatly benefited or arrested by quinia; if with Dr. Forry, and others, we admit that the subjection of these diseases to the same remedies which are found to arrest the course of the one, also arrests the course of the other, 'implies a close alliance, if not a common origin;' we shall be led to conclude, also, that all the other disorders in which it may be useful—wheresoever the locality, and at whatsoever season they may show themselves—are of malarial origin; and must, in like manner, take their appropriate place under the same plain designation. Such a mode of reasoning would lead us, if we wish to be consistent, to pathological deductions, at which our good sense must revolt, and wnich would ill accord with the principles of the inductive philosophy so dear to some of our opponents. No one will deny that mercury is the remedy for syphilis; for although, in the days of our infatuation for the Broussaian doctrine, many practitioners denied the necessity or propriety of that remedy, and attributed to it a thousand evils, experience has shown, and no less an authority than Ricord maintains, that it is superior to every other means, especially in the first stage of the disease, and that in many cases it cannot be superseded by any other. But mercury is found very useful, and even indispensable in various complaints; in hepatic and other glandular derangements; in inflammation of the serous membranes of the abdomen, chest, and head; in sundry diseases of the eye; iritis, for example. It is useful also in various other inflammations and engorgements, and even in some forms of periodic and malignant fevers. Surely, we shall look in vain for a pathologist disposed to conclude that the benefit derived in these latter diseases from mercury indicates their identity with, or dependence on syphilis, of which mercury must be viewed as the specific. If such an admission cannot be entertained—if we acknowledge that the advantages derived from mercury, in the diseases mentioned, in no way justify a belief in the identity of these with syphilis, it is difficult to perceive the propriety of viewing pneumonia as nothing more than a particular form of periodic fever, on the plea that quinia may prove useful when resorted to at the period of remission. The second limb of the argument, founded on treatment, must, therefore, like the first, be set aside."

Such is a pretty full account of the contents of this elaborate volume, of which we have already expressed the opinion, that it is entitled to take rank among the most meritorious of American medical productions. It is as scholarly in its style, as it is full in matter and powerful in argument. Not the least valuable feature in it is its constant reference to authorities, which renders it peculiarly interesting to those who wish to pursue researches in the same direction. If one is desirous of knowing what has been written on any of these subjects, he has only to turn to the pages of Dr. LA ROCHE. We cordially recommend his work to our readers as one which reflects honor upon the medical literature of our country.

